# Role of Depression and Anxiety in Eating Disorder Symptomatology in Higher Levels of Care for Girls With Anorexia Nervosa

**DOI:** 10.1002/jclp.70158

**Published:** 2026-06-09

**Authors:** Jasmine Ghannadpour, Savannah Dieste, Stephen Buerkert, Sunita M. Stewart

**Affiliations:** ^1^ Department of Psychiatry University of Texas Southwestern Medical Center Dallas Texas USA; ^2^ Department of Psychiatry and Psychology Children's Health Children's Medical Center Dallas Texas USA

**Keywords:** adolescent, anorexia, anxiety, depression, eating disorders, transdiagnostic treatment

## Abstract

**Objectives:**

Although the prevalence of affective comorbidities with eating disorders (ED) has been well established, the three‐way relation between ED, depression, and anxiety symptoms has not been explored in an intensive adolescent treatment setting.

**Methods:**

This study analyzed data from a sample of teenage girls with anorexia nervosa in higher levels of care (*n* = 457) to assess the relations between changes in ED symptoms, depression, and anxiety over the course of ED treatment.

**Results:**

This study demonstrated that improvements in depression and anxiety are significantly associated with improvement in ED symptoms.

**Conclusion:**

Findings support the importance of routine assessments for depression and anxiety among adolescents with anorexia nervosa in higher levels of care and a transdiagnostic approach to treatment that addresses shared underlying mechanisms. Future research that aims to uncover mediators and moderators of these relations is warranted to enhance clinical efforts.

## 1 Introduction

Anorexia nervosa (AN) has a lifetime prevalence of 0.1% to 3.6% in women and 0% to 0.3% in men (Van Eeden et al. 2021). Rates of AN have been steadily increasing for 10 to 14 year‐old girls (Petkova et al. 2019; Reas and Rø 2018). Specifically, girls are more likely to develop AN at an earlier age than boys, associated with trends in earlier onset of puberty in girls (Favaro et al. 2009; Gonzalez et al. 2007; Herpertz‐Dahlmann 2015; Swanson et al. 2011). Adolescents diagnosed with an eating disorder (ED), including those who eventually recover, have been found to be at increased risk for numerous biopsychosocial consequences; these include poorer physical health and higher rates of psychiatric comorbidities through early adulthood, such as personality disorders, substance use, and suicidality (Johnson et al. 2002).

Anxiety and depression are highly comorbid in adolescents (Brady and Kendall 1992; Seligman and Ollendick 1998), and specifically among adolescents who have been diagnosed with an ED (Blinder et al. 2006; Herpertz‐Dahlmann et al. 2001; Kaye et al. 2004; Laessle et al. 1987; Pollice et al. 1997; Salbach‐Andrae et al. 2008). However, implications of the co‐occurrence of ED, depression, and anxiety have not been thoroughly investigated. One study demonstrated that adolescent girls with AN who presented with comorbid depression and anxiety tended to have more severe ED symptoms—as well as more suicide attempts and hospitalizations—than girls with AN alone (Brand‐Gothelf et al. 2014). More severe depression and anxiety symptoms have also been associated with more severe ED symptoms (Sander et al. 2021), perhaps because the co‐occurrence of all three disorders may indicate a broader deficit in emotional regulation (Smith et al. 2018). Further supporting this claim, a recent systematic review found consistent associations between emotion regulation difficulties and EDs across the diagnostic spectrum, highlighting the transdiagnostic role of emotion regulation in ED psychopathology (Félix et al. 2025). Other common substrates amongst EDs, depression, and anxiety include general distress (Clark and Watson 1991) and perfectionism and self‐criticism (Egan et al. 2011; Williams and Levinson 2022).

Clinical implications of these comorbid symptoms warrant further investigation. Most studies investigating ED, depression, and anxiety symptoms are cross‐sectional or aimed at identifying predictors of ED; however, there is limited research on how these symptoms interact over the course of intensive treatment (i.e., inpatient and/or partial hospitalization). We propose a conceptual model such that ED, depression, and anxiety symptoms develop in parallel because of their shared substrates; therefore, ED treatment, which targets underlying vulnerabilities, results in parallel improvement in all symptoms. This rationale is supported by a recent review of progress and advancements in assessing and treating EDs in youth, which highlights overlapping symptomology as a key clinical challenge, proposes emotion regulation difficulties as a key mechanism linked to the development of EDs, and notes promising evidence for emerging approaches to ED treatment that target emotion regulation difficulties (Horovitz 2025).

It has been suggested that routine admission assessments are essential in identifying patients with higher symptomatology, who are at higher risk of poorer outcomes and dropping out of treatment (Vall and Wade 2015). A greater understanding of the role of affective comorbidities may inform enhancements to the treatment of AN among adolescent girls. This study aimed to assess the following in a sample of adolescent girls with AN in an intensive treatment program for EDs: (1) if depression and anxiety symptoms are associated with more severe ED pathology at admission and discharge, (2) how ED, depression, and anxiety symptoms change over the course of treatment independently; and (3) whether changes in depression and anxiety are associated with change in ED symptoms, with the hypothesis that internalizing symptoms improve in parallel with ED symptoms due to their shared underlying substrates.

## 2 Methods

### 2.1 The Context of the Study

Patients completed assessments upon admission into and discharge from adolescent ED inpatient or partial hospitalization programs as part of a program improvement initiative. The ED treatment program is guided by cognitive behavioral therapy, family‐based treatment, dialectical behavioral therapy, and acceptance and commitment therapy. Given high levels of comorbidity within the EDs population, a transdiagnostic approach is used in treatment, meaning that therapists target underlying mechanisms of EDs that are frequently present in depression and anxiety as well, such as distress intolerance, emotional dysregulation, perfectionism, interpersonal problems, and poor self‐esteem. Clinical interventions are developmentally informed (e.g., emotion identification with a faces sheet vs. a feelings wheel, more immediate positive reinforcement, play or activity‐based individual therapy). Expressive therapies, such as music, art, and recreational therapies, are also provided daily in the treatment program. Patients are seen twice weekly for family therapy and twice weekly for individual therapy.

Patients are typically discharged from the hospital when (1) weight restoration is at least 85% of target body weight, (2) they are able to maintain restored weight after a weekend at home, and (3) they are consistently eating most meals and snacks, as prescribed by their individual meal plans. Typical length of stay on inpatient is 2−3 weeks and on PHP is 4−6 weeks (approximate time between IP admission and PHP discharge: *n* = 217, *M* = 46.8 days, SD = 28.5 days). The majority of patients from the inpatient step‐down to PHP. Inpatient is a 12‐bed unit. PHP typically does not go above 15 but has some flexibility based on step‐down needs from the inpatient program. The census fluctuated throughout the time period observed. Each patient has a psychiatrist, family therapist, individual therapist, dietitian, and education specialist. Nurses and milieu therapists are also assigned to each child every day.

**Figure 1:**
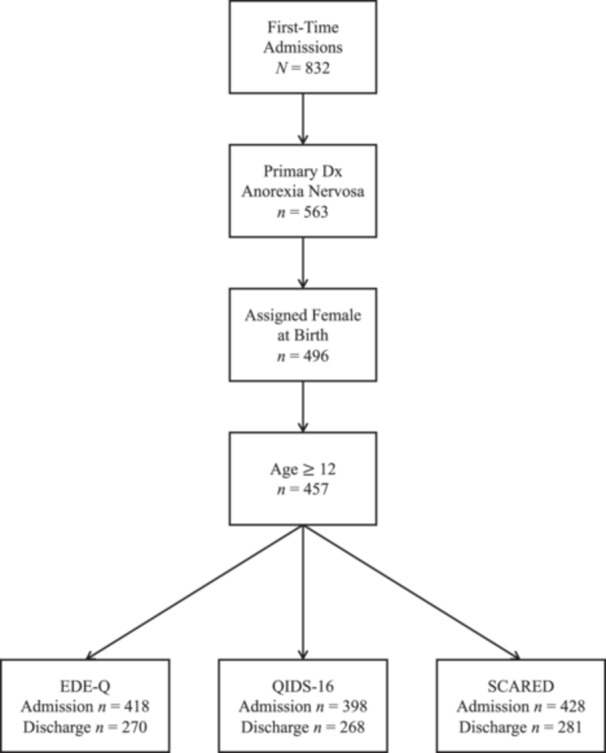
Sample selection flowchart.

Of the 832 first‐time admissions to the program on record between July 2018 and October 2024, 457 (54.9%) were assigned female at birth, aged 12 to 18 (*M* = 14.5 ± 1.6 years), and had a primary diagnosis of AN. Patients who were males and those with other primary ED diagnoses (e.g., bulimia nervosa, avoidant/restrictive food intake disorder, and atypical AN) were excluded to maintain homogeneity of the sample. Patients in the sample had an average length of stay of 46.8 (±28.5) days (*n* = 276). As is common in studies conducted in clinical settings that collect multiple data points, not every patient completed each assessment at both admission and discharge (Figure 1). Sample demographics and baseline characteristics are presented in Table 1.

**Table 1:** Sample demographics and baseline clinical characteristics.

*n* = 457^a^		
Variable/Category	Mean or *n*	SD or %
Age	14.52	1.56
Length of stay (days)^b^	46.78	28.53
*Race*		
White or caucasian	404	88.4%
Asian	34	7.4%
Black or African American	12	2.6%
Two or more races	7	1.5%
*Ethnicity*		
Non‐Hispanic	276	60.4%
Hispanic	181	39.6%
*EDE‐Q (eating disorder)*		
At/above cutoff (≥4)	203	44.4%
Below cutoff (<4)	254	55.6%
*QIDS‐16‐SR (depression)*		
Very severe (21–27)	32	7.0%
Severe (16–20)	108	23.6%
Moderate (11–15)	118	25.8%
Mild (6–10)	121	26.5%
Minimal or none (0–5)	77	16.8%
*SCARED (anxiety)*		
At/above cutoff (≥25)	356	77.9%
Below cutoff (<25)	101	22.1%

^a^ After multiple imputation.

^b^ Length of stay *n* = 276.

### 2.2 Measures

#### 2.2.1 ED Examination (EDE)–Questionnaire (EDE‐Q)

The EDE‐Q (Fairburn and Beglin 2008) is a 28‐item self‐report measure of ED psychopathology, adapted from the ED Examination (EDE; Cooper and Fairburn 1987). The assessment measures ED symptoms within the last 28 days and is often used to measure change in symptoms over the course of treatment (Rose et al. 2013). The EDE‐Q is scored using a seven‐point Likert scale (ranging from zero to six) and provides a global score (average of four subscales containing 22 items total). A commonly used clinical cut‐off for the global EDE‐Q score is equal to or greater than 2.8 (Velkoff et al. 2023). The EDE‐Q has shown good internal consistency with female adolescents with AN (*α* = 0.96 for global score; Jennings and Phillips 2017). Similarly, the EDE‐Q showed good reliability in our sample at admission (22 items, *n* = 418, *α* = 0.97) and discharge (22 items, *n* = 270, *α* = 0.97).

#### 2.2.2 Quick Inventory of Depressive Symptomatology–Self Report (QIDS‐16‐SR)

The QIDS‐16‐SR (Rush et al. 2006; Rush et al. 2003) is a 16‐item self‐report questionnaire which measures the presence and severity of depression symptoms. Adolescents respond to items on a four‐point Likert scale (0–3), with higher total scores (sum of nine items) indicating more severe depression symptoms. Total score thresholds for depression severity are as follows: 0–5 (minimal or no depression), 6–10 (mild depression), 11–15 (moderate depression), 16–20 (severe depression), 21–27 (very severe depression). The threshold for meaningful change is 3.5 points (McIntyre et al. 2021). The QIDS‐16‐SR has demonstrated good reliability in adolescents (*α* = 0.80; Bernstein et al. 2010), which was upheld in our sample at both admission (9 items, *n* = 398, *α* = 0.85) and discharge (9 items, *n* = 268, *α* = 0.79).

#### 2.2.3 Screen for Child Anxiety Related Emotional Disorders (SCARED)

The SCARED (Birmaher et al. 1997) is a 41‐item self‐report inventory rated on a three‐point Likert scale (ranging from zero to two), which screens for symptoms of anxiety disorders in children and adolescents. Total scores (sum of all 41 items) of 25 or above may indicate the presence of an anxiety disorder. The SCARED has shown good internal consistency in the literature (*α* = 0.74 to 0.93; Birmaher et al. 1999). Likewise, the SCARED showed good reliability in our sample at admission (41 items, *n* = 428, *α* = 0.94) and discharge (41 items, *n* = 281, *α* = 0.95).

### 2.3 Data Analysis

SPSS Statistics v27 was used for statistical procedures and analyses.

Values for total scores were 9.3% (127/1371) and 40.3% (552/1371) missing at admission and discharge, respectively. For these total scores, Little's MCAR test was nonsignificant (*χ* ^2^(57) = 55.47, *p* = 0.533), so we multiply imputed missing values by fully conditional specification (*m* = 100). Length of service and age served as supplemental predictors during imputation.

For our first aim, examining whether internalizing symptoms (depression and anxiety) are associated with more severe ED pathology, a correlation analysis was conducted on total scores at admission and discharge.

For our second aim, which sought to identify changes in depression, anxiety, and ED symptoms over the course of treatment, paired‐samples *t* tests were used to test the change in total scores between admission and discharge (Table 2).

**Table 2:** Pearson correlations and paired‐samples *t* tests.

*n* = 457 variable/Statistic	EDE‐Q (eating disorder)	QIDS‐16‐SR (depression)	SCARED (anxiety)
EDE‐Q		0.68***	0.56***
QIDS‐16‐SR	0.67***		0.62***
SCARED	0.56***	0.65***	
Admission *M*, (SD)	3.25 (1.79)	11.92 (5.73)	37.73 (16.50)
Discharge *M*, (SD)	2.60 (1.60)	10.52 (4.99)	36.77 (17.02)
*t* (df)	−7.64 (1067.05)	−4.91 (961.01)	−1.39 (779.60)
*d* a	−0.43***	−0.28***	−0.08

*Note:* Upper‐right triangle Pearson correlations are at discharge.

*** *p* < 0.001.

^a^ Calculated using the MI‐pooled standard deviation of the mean difference.

For our third aim, we used generalized estimating equations (GEE) models to examine the relationships between the changes in ED, depression, and anxiety symptoms from admission to discharge when controlling for the effect of treatment. GEE is commonly used for regression analysis of data exhibiting dependence between observations, such as is the case with repeated measures, wherein dependent observations can be clustered by the individual to whom they belong (Austin et al. 2024; Liang and Zeger 1986). To separate the effect of the time‐invariant correlations between scales from the effect of the change over time between scales, each scale's total score was averaged and centered at the person level, providing two predictors per scale for use in analyses: a person‐mean score and a person‐mean centered score (Wang and Maxwell 2015).

We first examined relationships between two scales at a time while controlling for treatment. For each of the three scale total scores as the DV, two linear GEE models were used. Each model included a binary factor for time point—to measure and control for the effect of treatment—in addition to the person‐mean and person‐mean‐centered score of the predicting scale as the IVs (i.e., two covariates and one binary factor as IVs per model). For the sake of comparison, we included the GEE equivalents of each of the three *t* tests used in our second aim (i.e., one binary factor as the IV per model).

We then examined relationships between all three scales at a time when controlling for treatment. For each of the three scale total scores as the DV, one linear GEE model was used. Each model contained two scales as IVs via their person‐mean and person‐mean‐centered scores in addition to a binary factor for time point as predictors (i.e., four covariates and one binary factor per model). As length of stay is a between‐subjects covariate and would not influence our within‐subjects effects of interest if included as a main effect, we did not include it in our original analyses.

Note that because the person‐mean centered scores were calculated as the distance of admission and discharge scores from their midpoint (half the arithmetic difference between admission and discharge), the unstandardized regression coefficients (*B*) corresponding to these centered scores are in units of “point change in the DV scale per 0.5‐point change in the IV scale” (i.e., a 1‐point change in the IV scale from admission to discharge predicted a change in the DV scale across the same time frame equal to *B* × 2). All GEE models used to test our third aim specified an AR(1) working correlation matrix structure to handle the dependence of observations and used robust estimation of covariance to reduce the bias potentially introduced by assumption violations such as heteroscedasticity.

## 3 Results

Aim 1 analyses indicated all variables were significantly correlated within each time point, such that more severe ED symptoms were associated with more severe depression and anxiety symptoms at both admission and discharge (Table 2).

Aim 2 analyses found ED and depression symptoms significantly decreased over the course of treatment. Although anxiety symptoms also decreased between admission and discharge, the observed change was marginal in effect size and not statistically significant. The average change in ED symptoms corresponded to a transition from 0.5 points above the global cutoff (2.8) to 0.2 points below it, while the average change in depression symptoms (−1.4) corresponded to 40% of the threshold of clinically meaningful change (3.5).

Aim 3 analyses reported changes in ED, depression, and anxiety symptoms as significantly correlated (Table 3, models 1‐3b‐c), such that improvements in ED, depression, and anxiety symptoms all significantly predicted improvements in each other when examined pairwise and controlling for treatment. This also held true when controlling for the effect of the change in the remaining scale in each of the final three GEE models. Notably, while previous paired‐samples *t* tests indicated a significant effect of treatment on depression symptoms, this effect fell out of significance when controlling for the change in ED symptoms. However, when controlling for both the change in anxiety symptoms and the change in ED symptoms, the effect of treatment on depression symptoms once again became significant. Also of note was the effect of treatment on anxiety symptoms in the presence of controls. When controlling for the change in both depression and ED symptoms, the nonsignificant effect of treatment on improving anxiety diminished such that, in the absence of improvement in both ED and depression symptoms, anxiety was predicted to marginally worsen over treatment.

**Table 3:** GEE Models of ED (EDE‐Q), depression (QIDS‐16‐SR), and anxiety (SCARED)—effects of treatment, Person‐mean (M_p_), and Person‐mean centered (C_p_) symptom scores (*n* = 457).

Model	DV	IV	*B*	95% CI	β	*p*
1a	ED	(Intercept)	3.25	[3.08–3.42]	—	< 0.001
		Treatment^a^	−0.65	[−0.82 to −0.48]	—	< 0.001
1b	ED	(Intercept)	0.60	[0.33–0.87]	0.12	< 0.001
		Depression (M_p_)^b^	0.23	[0.20–0.25]	0.71	< 0.001
		Treatment	−0.42	[−0.57 to −0.27]	−0.24	< 0.001
		Depression (C_p_)^c^	0.17	[0.13–0.20]	0.52	< 0.001
1c	ED	(Intercept)	1.19	[0.86–1.51]	0.17	< 0.001
		Anxiety (M_p_)	0.05	[0.05–0.06]	0.53	< 0.001
		Treatment	−0.58	[−0.73 to −0.44]	−0.34	< 0.001
		Anxiety (C_p_)	0.07	[0.06–0.08]	0.68	< 0.001
1d	ED	(Intercept)	0.37	[0.09–0.66]	0.14	< 0.05
		Depression (M_p_)	0.19	[0.16–0.22]	0.59	< 0.001
		Anxiety (M_p_)	0.02	[0.01–0.03]	0.18	< 0.001
		Treatment	−0.47	[−0.62 to −0.32]	−0.27	< 0.001
		Depression (C_p_)	0.10	[0.06–0.14]	0.32	< 0.001
		Anxiety (C_p_)	0.04	[0.02–0.06]	0.41	< 0.001
2a	Depression	(Intercept)	11.92	[11.38–12.46]	—	< 0.001
		Treatment	−1.40	[−1.96 to −0.84]	—	< 0.001
2b	Depression	(Intercept)	4.84	[4.08–5.61]	0.02	< 0.001
		ED (M_p_)	2.22	[1.99 – 2.44]	0.71	< 0.001
		Treatment	−0.22	[−0.74 to 0.30]	−0.04	ns
		ED (C_p_)	1.81	[1.48–2.14]	0.58	< 0.001
2c	Depression	(Intercept)	4.62	[3.77–5.48]	0.11	< 0.001
		Anxiety (M_p_)	0.19	[0.17–0.21]	0.60	< 0.001
		Treatment	−1.14	[−1.59 to −0.69]	−0.21	< 0.001
		Anxiety (C_p_)	0.27	[0.23–0.31]	0.84	< 0.001
2d	Depression	(Intercept)	2.92	[2.16–3.68]	0.06	< 0.001
		ED (M_p_)	1.60	[1.33–1.87]	0.51	< 0.001
		Anxiety (M_p_)	0.10	[0.08–0.13]	0.32	< 0.001
		Treatment	−0.60	[−1.07 to −0.13]	−0.11	< 0.05
		ED (C_p_)	0.93	[0.58–1.28]	0.30	< 0.001
		Anxiety (C_p_)	0.21	[0.16–0.25]	0.64	< 0.001
3a	Anxiety	(Intercept)	37.73	[36.18–39.28]	—	< 0.001
		Treatment	−0.96	[−2.32 to 0.40]	—	ns
3b	Anxiety	(Intercept)	19.23	[16.24–22.21]	−0.05	< 0.001
		ED (M_p_)	5.85	[4.97–6.72]	0.60	< 0.001
		Treatment	1.82	[0.50–3.15]	0.11	< 0.01
		ED (C_p_)	4.27	[3.39–5.16]	0.44	< 0.001
3c	Anxiety	(Intercept)	13.08	[9.89–16.26]	−0.03	< 0.001
		Depression (M_p_)	2.10	[1.84–2.37]	0.68	< 0.001
		Treatment	1.16	[0.05–2.28]	0.07	< 0.05
		Depression (C_p_)	1.52	[1.26–1.77]	0.49	< 0.001
3d	Anxiety	(Intercept)	11.69	[8.62–14.75]	−0.06	< 0.001
		ED (M_p_)	2.38	[1.15–3.61]	0.24	< 0.001
		Depression (M_p_)	1.56	[1.17–1.96]	0.51	< 0.001
		Treatment	2.08	[0.91–3.26]	0.12	< 0.001
		ED (C_p_)	2.19	[1.28–3.10]	0.23	< 0.001
		Depression (C_p_)	1.15	[0.88–1.42]	0.37	< 0.001

*Note:* Given t_1_ = admission score and t_2_ = discharge score.

^a^ Treatment—The effect of treatment on DV at t_2_ with respect to t_1_. Negative coefficients (*B* and β) indicate improvement. Equivalent to a paired‐samples *t* test in models 1‐3a.

^b^ M_p_—Person‐mean score. Calculated for each individual as the average of the predicting scale at t_1_ and t_2_. Positive coefficients indicate a positive time‐invariant correlation between the predicting scale and DV.

^c^ C_p_—Person‐mean centered score. Calculated for each individual as [t_i_−M_p_]. Positive coefficients indicate a positive correlation between changes in both the IV and DV. E.g., in model 1b, a change of −1 point in depression predicts a change of −0.34 points in ED (where −0.34 = *B* × 2, as centered scores C_p_ are measured as distance of the IV at t_1_ and t_2_ from M_p_, their midpoint).

To provide an approximate comparison of the symptom‐derived effects found in the largest GEE models (Table 3, models 1−3d): when controlling for the effect of treatment, the first of the largest GEE models indicated that improvements in depression and anxiety symptoms predicted improvements in ED symptoms to a similar extent. However, the extent to which depression and anxiety symptoms predicted ED symptoms, ignoring change over time (i.e., the prediction by the person‐mean scores) was noticeably different, with depression symptoms being the stronger predictor of ED symptoms. The second of the largest GEE models indicated that the prediction of change in depression symptoms by the change in ED symptoms was less substantial than the prediction by the change in anxiety symptoms. When ignoring change over time, the prediction of depression symptoms by ED symptoms and of depression symptoms by anxiety symptoms were comparable. In the third of the largest GEE models, for both time‐variant and ‐invariant effects, the prediction of anxiety symptoms by depression symptoms was stronger than that of the prediction of anxiety symptoms by ED symptoms.

## 4 Discussion

For adolescent girls diagnosed with AN in higher levels of care, symptom severity of ED, depression, and anxiety were all positively correlated, such that more severe ED symptoms were associated with more severe anxiety and depression symptoms, consistent with current literature (Brand‐Gothelf et al. 2014; Sander et al. 2021). This finding supports the importance of providers routinely assessing for depression and anxiety in higher levels of ED treatment and tailoring treatment to potentially address all three sets of symptoms. An argument could also be made that adolescent girls in higher levels of care for affective disorders should be screened for EDs. Of note, one study found many adolescents with an ED engage in mental health care for emotional concerns, including affective disorders, but most never seek treatment specifically for their ED (Swanson et al. 2011). This points to generalized outpatient mental health care as an important potential place of targeted intervention for adolescents struggling with ED symptomatology, who may be more willing or comfortable discussing ED‐related concerns with their existing mental health professionals.

Our study also found that changes in depression and anxiety symptoms are significantly related to changes in ED symptoms, even when controlling for treatment and the effective change of the third/remaining scale. In line with two prior adult studies which found that reduction in depression symptoms (Bäck et al. 2020) and anxiety symptoms (Velkoff et al. 2024) predicted reduction in ED symptoms, our results suggest that clinicians could incrementally predict how much ED symptoms improve based on improvement in symptoms of both depression and anxiety. Although the paired‐samples *t* tests showed significant improvement only in ED and depression symptoms over the course of treatment, the GEE models indicated that the improvements in both depression and anxiety were significantly associated with improvement in ED symptoms over the course of treatment. Therefore, even a relatively small improvement in anxiety symptoms may be associated with a significant improvement in ED symptoms. It is possible that premorbid trait anxiety could be harder to treat, and we have also observed that stress towards the end of hospitalization could be associated with facing the demands of returning home and school and leaving the support of the hospital setting. Our study also found that change in ED symptoms accounted for the significant positive effect of treatment on depression symptoms. However, depression became significant again when both anxiety and ED symptom changes were controlled, suggesting that the effects of treatment on depression improvement may be mediated by or moderated by changes in ED and anxiety symptoms. Moreover, change in either or both depression and ED symptoms accounted for the nonsignificant but positive effect of treatment on anxiety symptoms, suggesting that anxiety symptoms may not improve over treatment unless ED or depression symptoms also improve. These findings highlight the need for clinicians to target underlying factors spanning across all three symptom domains to address them in parallel, and warrant future studies which uncover mediators driving these effects and moderators facilitating or inhibiting positive changes. Along these lines, a recent review (Wade et al. 2024) presents a protocol for managing co‐occurring mental health conditions in treatment for EDs, proposing sequential or adaptive treatment designs as optimal approaches. Future research might employ such adaptive treatment designs or dismantling trials to elucidate whether targeting depression or anxiety yields incremental benefits in ED treatment outcomes.

The effects demonstrated by our model suggest that changes in anxiety and depression predict parallel changes in ED symptoms, perhaps due to their shared underlying substrates targeted during ED treatment. These findings align with prior studies among adolescents which found that symptoms of anxiety (Trompeter et al. 2025) and depression (Kenny et al. 2022; Sharpe et al. 2018) are prospectively and bi‐directionally linked to ED symptoms. Our study adds to the literature by demonstrating that these relationships are upheld in clinical youth in a higher level of care, and when examining symptom improvement rather than symptom worsening. This information might be used to inform not only risk assessment but also transdiagnostic treatments that target depression and anxiety alongside ED symptoms. For example, the transdiagnostic model of family‐based treatment for adolescents with EDs (Loeb et al. 2012) may be particularly effective, as this approach has shown efficacy in reducing comorbid depression and anxiety (Trainor et al. 2020). Recent trials of the Unified Protocol in adolescent populations provide further empirical support for transdiagnostic approaches which target underlying emotional symptoms as acceptable and effective treatments for comorbid psychological disorders (Liu et al. 2025; Peláez et al. 2024), Our study also found differences in the extent to which changes in depression and anxiety symptoms predicted changes in ED symptoms, and vice versa, depending upon whether or not change over time was ignored, suggesting that treatment effects influence the strength of these predictions. Future investigations are needed to pinpoint the relative magnitude of these changes in terms that translate into clinical care (e.g., cutoff scores).

Although we accounted for age and LOS, it is possible that other confounding variables may be influencing changes in symptoms over the course of treatment. For example, a prior study found that depression and anxiety symptoms at least partially decreased with nutritional rehabilitation (Mattar et al. 2012). Depression and anxiety are also strongly related to rumination and avoidance, both of which could also strongly influence ED symptoms (Aldao et al. 2010). Since treatment techniques used for youth with EDs can and often overlap with common methods of treating depression and anxiety (e.g., increasing adaptive coping skills, identifying relations between thoughts, emotions, and behaviors, identifying rational thoughts), it is not surprising that the treatment provided in the current study resulted in significant improvement in a number of primary ED and comorbid symptoms.

This study also provides normative clinical information regarding an adolescent ED population. This information could be helpful in identifying patients who may be underreporting ED symptoms, such as those whose scores are significantly below average. Although adolescents with AN tend to minimize or deny ED symptoms (Couturier and Lock 2006), it is possible that girls who have reached the need for a higher level of care for EDs are less likely to do so.

Limitations of this study include the absence of parent reports, which may have limited our ability to capture a more comprehensive, multi‐informant understanding of functioning, and the absence of a control group, which prevented us from determining whether the observed symptom changes occurred over time regardless of hospitalization and treatment, an effect known as regression to the mean. However, the collection of assessment measures at admission and discharge allowed us to control for time as a proxy for treatment effects. In addition, anxiety and depression were not individually experimentally manipulated; therefore, the influence of improvement in these symptoms as a driver of change could not be determined. One researcher has suggested that anxiety and ED are so closely related that the early treatment of anxiety symptoms could prevent an ED (Hocaoglu 2017), and the same may be true for depression. Future randomized controlled trials, which have the experimental manipulation and randomization necessary to infer causality, are warranted. Our study is also limited by the use of self‐report measures for all symptoms, which are subject to participant biases. While our relatively large sample size is a strength of this study, we lacked sufficient power to examine potential mediators and moderators of these changes. Lastly, these findings are specific to a single adolescent ED program and may not be generalizable to other programs that target symptoms of depression and anxiety differently. Despite these limitations, we believe our study adds to the current ED literature.

Future studies that more specifically pinpoint direct mechanisms of change in ED treatment are warranted to increase the field's understanding of how to maximize positive treatment outcomes while shortening LOS. These findings also support further exploration of the presence of other psychological concerns related to ED symptoms (e.g., perfectionism and poor self‐image) and the impact of treating these in conjunction with ED symptoms alone. Importantly, this study provides evidence to support not only the implementation of routine assessments for depression and anxiety in treatment programs for adolescent EDs, but also transdiagnostic approaches to care which target internalizing symptoms in tandem with ED symptomology.

## Conflicts of Interest

The authors declare no conflicts of interest.

## Data Availability

The data that support the findings of this study are available on request from the corresponding author. The data are not publicly available due to privacy or ethical restrictions.
